# Fluorescence-guided surgery combined with intraoperative photodynamic therapy for recurrent atypical and anaplastic intracranial meningiomas: a prospective feasibility study

**DOI:** 10.3389/fonc.2026.1767269

**Published:** 2026-02-25

**Authors:** Anastasiia Nechaeva, Konstantin Kukanov, Alexey Ulitin, Victor Olyushin, Daria Sitovskaya, Danila Bobkov, Vseslav Ushanov, Stephanie E. Combs, Konstantin Samochernykh, Maxim Shevtsov

**Affiliations:** 1 Polenov Russian Research Neurosurgical Institute, Almazov National Medical Research Centre, St. Petersburg, Russia; 2 Laboratory of Biomedical Nanotechnologies, Institute of Cytology of the Russian Academy of Sciences (RAS), St. Petersburg, Russia; 3 Department of Radiation Oncology, Klinikum rechts der Isar, Technical University of Munich, Munich, Germany

**Keywords:** 5-aminolevulinic acid, anaplastic meningioma, atypical meningioma, fluorescence imaging, fluorescence-guided surgery, photodynamic therapy, recurrent meningioma

## Abstract

**Objective:**

Recurrent intracranial meningiomas are a significant therapeutic challenge due to their invasive growth and high recurrence risk after surgery and radiotherapy. This study investigates the feasibility of a novel integrated approach combining 5-aminolevulinic acid (5-ALA) fluorescence-guided surgery (FGS) with intraoperative photodynamic therapy (PDT) for recurrent atypical and anaplastic meningiomas.

**Methods:**

In a single-center, prospective cohort study, 23 patients with recurrent atypical and anaplastic meningiomas received the experimental treatment protocol (FGS+PDT). A retrospective control group (n=35) underwent conventional microsurgery. The intervention included preoperative 5-ALA administration, FGS with visual (Fluorescence Intensity Score, FIS) and quantitative biospectroscopy (Fluorescence Index, FI) guidance, tumor resection, and subsequent PDT (635 nm laser) applied to the resection cavity and tumor matrix. Biospectroscopy guided PDT endpoint (photobleaching and decreasing of FI). Primary outcomes included feasibility, safety, and extent of resection (Simpson Grade), short follow-up period. Histopathological and immunofluorescence analyses of paired pre-/post-PDT biopsies assessed biological effects.

**Results:**

The FGS+PDT protocol was successfully completed in all patients with an excellent safety profile; no adverse events were attributed to 5-ALA or PDT. All tumors exhibited visible 5-ALA fluorescence. Gross-total resection (Simpson I-II) was achieved in 95.6% (22/23) of the study group versus 77.1% (27/35) in controls (p<0.05). Biospectroscopy revealed significant PpIX accumulation even in visually low-fluorescence tumors. Over a median follow-up of 16 months, no recurrences were observed in the experimental group. Histopathological analysis demonstrated profound PDT-induced effects, including total ablation of progesterone receptor expression in the tumor matrix and a significant increase in caspase-3-mediated apoptosis in the peritumoral zone (36.3 ± 9.6 vs. 14.8 ± 2.2 cells/mm², p<0.0001). Confocal microscopy confirmed subcellular damage, including mitochondrial dysfunction, nuclear degradation, and Hsp70 overexpression.

**Conclusion:**

The integrated FGS and PDT protocol is feasible, safe, and demonstrates compelling preliminary efficacy for recurrent atypical and anaplastic meningiomas. It enhances resection and induces profound cytotoxic and apoptotic effects in the residual tumor bed and peritumoral zone. These results need to be further validated in larger, randomized controlled trials.

## 1 Introduction

Meningiomas are the most commonly occurring CNS tumors in adults (42.6% of all tumors) (1). The overall proportion of WHO grades 1, 2, and 3 intracranial meningiomas was 94.6%, 4.2%, and 1.2%, respectively (2); however, these data may differ across studies. Tumor recurrence represents a major challenge in the management of meningioma patients (3–8). The main prognostic factors are the extent of surgical resection and meningioma grade (2, 7). Favorable outcomes are typically achieved with radical resection (i.e., in non-skull base meningiomas without infiltration of nerves, vessels, dural venous sinuses or bone) and in benign meningiomas treated with surgery and radiotherapy. The overall rate of meningioma recurrence is 14-33% according to different authors, and it can occur despite treatment with total resection and subsequent radiotherapy (3–8). The biological aggressiveness of meningiomas, defined by their locally destructive growth that precludes radical resection, involves invasion of the dura mater, brain, blood vessels, dural venous sinuses, skull bones, and is more commonly associated with grade 2 and 3 tumors (9, 10). The 5-year recurrence rate is 0-2.36% for grade 1 meningiomas and 7.35-11.46% for grade 2 meningiomas. In contrast, grade 3 meningiomas have the most aggressive behavior, demonstrating a recurrence rate of 50–94% (5).

Recurrences predominantly are located within or near the surgical bed (11). Pharmacotherapy can be used in meningioma, especially in malignant and other recurrent tumors; however, no effective standard pharmacological regimens are established for these patients (2, 7, 12). Therefore, local tumor control represents a critical focus in the meningioma clinical management. Despite being the primary modalities for tumor local control, both surgical resection and radiosurgery demonstrate suboptimal effectiveness against malignant and recurrent meningiomas (2, 6–9). Therefore, there is a pressing clinical need to develop and refine intraoperative local therapeutic strategies that can improve the radicality of resection for atypical and anaplastic meningiomas while simultaneously targeting residual tumor cells. Photodynamic therapy represents a promising modality for the local control of meningiomas.

Photodynamic therapy is a modern oncological treatment; however, its clinical application in neuro-oncology is still at the clinical study stage, primarily for gliomas and less frequently for meningiomas (13–17). PDT was developed as an adjuvant to surgery to improve its efficacy via a photochemical reaction, which relies on three components: a photosensitizer (PS), light, and oxygen. The antitumor effect of PDT involves three key mechanisms: direct cell death induced by reactive oxygen species (ROS), vascular damage, and the induction of an acute inflammatory response that mobilizes the immune system (18). Furthermore, some PSs, such as 5-aminolevulinic acid (5-ALA), are also used in intraoperative fluorescence diagnostics. Research on the application of PDT for meningiomas is scarce and predominantly based on cell line studies. Nonetheless, these preliminary investigations demonstrate the feasibility of this approach (19–22).

5-Animolevulinic acid (5-ALA) is one of the most useful photosensitizers for intraoperative application. It is a highly selective theranostic agent with a short biological half-life (14). The rationale for using 5-ALA in PDT lies in its role as a precursor in heme biosynthesis. Following exogenous administration, 5-ALA is metabolized to the photoactive compound protoporphyrin IX (PpIX). Under normal conditions, PpIX is converted to heme by ferrochelatase in an iron-dependent reaction. However, the limited availability of iron and reduced ferrochelatase activity in tumor cells leads to selective PpIX accumulation, enabling PDT. PpIX accumulation in meningioma cells upon 5-ALA administration has been reported in several studies (23, 24). Additionally, *in vitro* experiments confirmed the cytotoxic efficacy of 5-ALA-based photodynamic therapy against meningioma cell lines (20, 21). This PS is particularly valuable for visualizing superficially located infiltrative tumor parts and residual tissue during surgery. However, visual assessment of 5-ALA-induced PpIX fluorescence intensity is inherently subjective and operator-dependent. This limitation underscores the necessity for implementing objective methods to quantify PpIX accumulation intraoperatively.

There is no established single standard for brain tumors PDT in modern guidelines and scientific works, and no studies have investigated intraoperative fluorescence diagnostics and PDT in recurrent meningiomas. Thus, we conducted a prospective, single-center pilot study to evaluate the feasibility of combined fluorescence-guided surgery and photodynamic therapy for the treatment of in recurrent intracranial atypical and anaplastic meningiomas.

## 2 Methods

### 2.1 Study design and participants

This single-center, prospective, open-label feasibility cohort study was conducted at the Department of Neurosurgery No. 4 of the Polenov Russian Research Neurosurgical Institute (Almazov Medical Research Centre). Study protocol was approved by the Ethics Committee of the Almazov Medical Research Centre (approval No. 0312–22 from 26 December 2022) and was conducted in accordance with the Declaration of Helsinki. Written informed consent was obtained from all participants. All studies were conducted in compliance with applicable guidelines and regulations.

Experimental group (prospective cohort) of 23 patients with recurrent atypical and anaplastic meningiomas was enrolled between March 2023 and October 2024. Control Group (retrospective cohort) of 35 patients was identified by reviewing the medical records of patients who had undergone conventional microsurgical resection (without fluorescence guidance or PDT) of recurrent intracranial meningioma at our institution between January 2020 and February 2023. These patients were selected to match the inclusion criteria of the prospective cohort as closely as possible. As our study focused on a novel combined intervention (5-ALA-guided surgery and PDT) for highly aggressive, recurrent atypical and anaplastic meningiomas, a randomized controlled design was deemed ethically challenging at this stage. Given the limited therapeutic options for these patients, we prioritized assessing the safety and preliminary efficacy of the combined modality while utilizing a historical cohort for comparison. All surgical procedures in both cohorts were performed by the same team of neurosurgeons at a single high-volume center, ensuring consistency in surgical philosophy and technique over time. Inclusion criteria: adult patients (age ≥ 18 years) with a radiologically confirmed diagnosis of recurrent atypical or anaplastic intracranial meningioma (according to the WHO 2021 classification (9);) based on preoperative magnetic resonance imaging (MRI); the recurrent tumor was deemed suitable for surgical intervention by the multidisciplinary tumor board; Karnofsky Performance Status (KPS) ≥ 70. Exclusion criteria: patients with known porphyria or severe hypersensitivity to porphyrins; severe hepatic or renal dysfunction; pregnancy or lactation; concomitant oncological diseases (except for radically treated skin cancer *in situ* more than 5 years ago); inability to undergo preoperative MRI with contrast enhancement.

To ensure the comparability of the cohorts, we performed a detailed analysis of baseline characteristics (**Table 1**), which confirmed that the groups were well-matched for key prognostic factors.

**Table 1:** Baseline demographic and clinical characteristics of the patients cohort.

Demographic and clinical characteristics	Study group n=23	Control group n=35	P-value (comparison between groups)
Age (years)			
Mean ±SD	61.3±10.2	67.5±8.1	p=0.48
Sex, n (%)			
Female	14 (60.9)	20 (57.2)	p=0.79
Male	9 (39.1)	15 (42.8)	
KPS at presentation, n (%)			
70-80	16 (69.5)	24 (68.6)	p=0.93
90-100	7 (30.5)	11 (31.4)	
WHO grade at initial diagnosis, n (%)			
II	17 (73.9)	25 (71.4)	p=0.83
III	6 (26.1)	10 (28.6)	
WHO grade at recurrence, n (%)			
II	14 (60.9)	22 (62.8)	p=0.87
III	9 (39.1)	13 (37.2)	
Number of prior surgeries, n (%)			
1	9 (39.1)	14 (40.0)	p>0.05
2	8 (34.8)	11 (31.4)	
≥ 3	6 (26.1)	10 (28.6)	
History of prior radiotherapy, n (%)			
Yes	9 (39.1)	15 (42.8)	p=0.77
No	14 (60.9)	20 (57.2)	
Tumor location, n (%)			
Skull Base	13 (56.5)	18 (51.4)	p=0.79
Convexity	10 (43.5)	17 (48.6)	
Preoperative MRI peritumoral edema, n (%)			
Present	16 (69.5)	22 (62.8)	p=0.77
Absent	7 (30.5)	13 (37.2)	
Preoperative dexamethasone therapy, n (%)			
Yes	1 (4.4)	2 (5.7)	p=0.81
No	22 (95.6)	33 (94.3)	
Lymphocytic infiltration, n (%)			
Present	12 (52.2)	18 (51.4)	p=0.95
Absent	11 (47.8)	17 17 (48.6)	
Proliferation of microvessels (Angiomatosis), n (%)			
Present	20 (86.9)	31 (88.6)	p=0.85
Absent	3 (13.1)	4 (11.4)	
Ki-67 proliferation index, n (%)			
≤ 9%	14 (60.9)	22 (62.8)	p=0.87
> 9%	9 (39.1)	13 (37.2)	
VEGF expression, n (%)			
Low	18 (78.3)	27 (77.1)	p=0.92
High	5 (21.7)	8 (22.9)	

### 2.2 Intervention protocol

**Figure 1:**
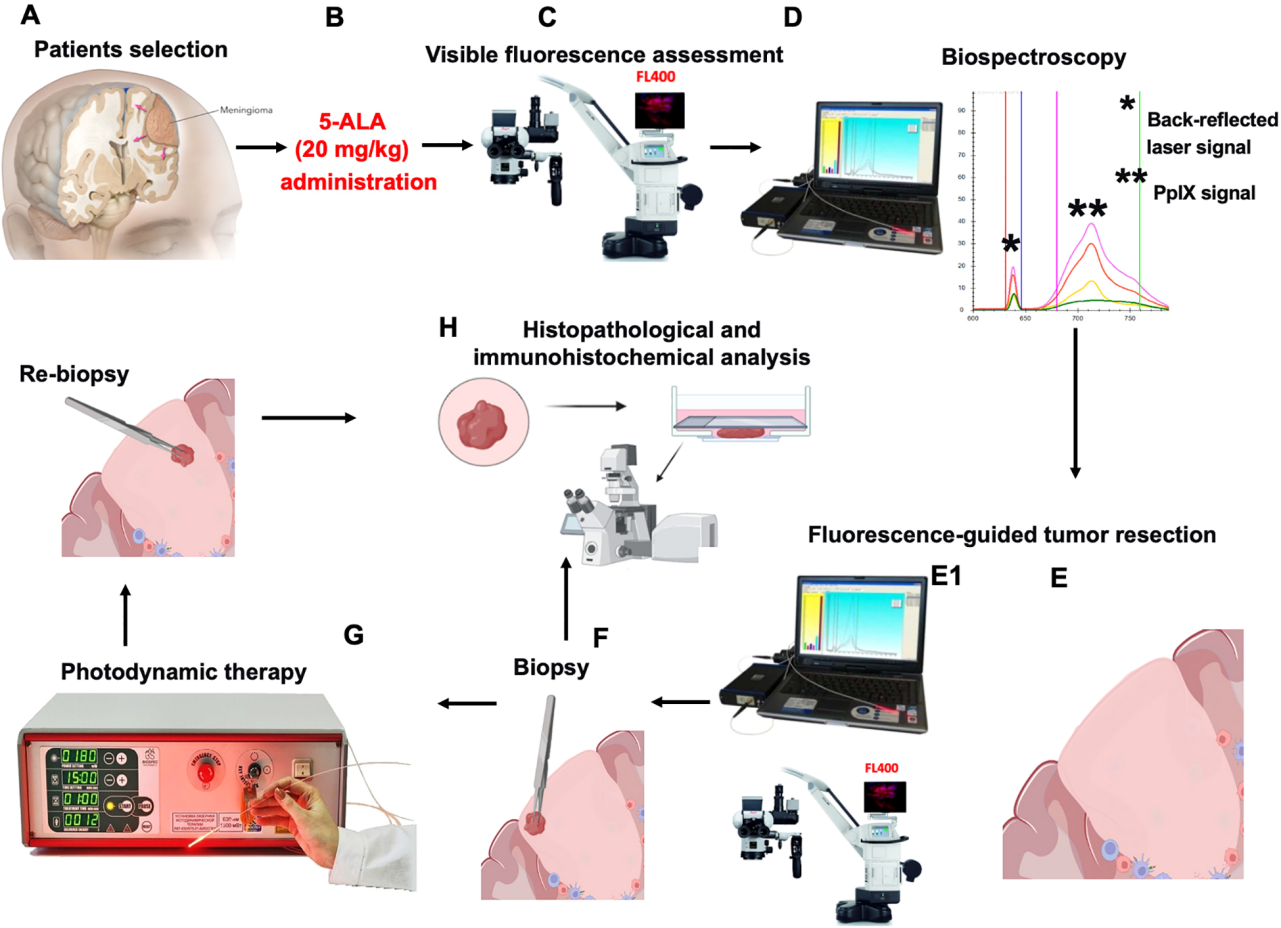
Schematic of the combined fluorescence-guided surgery and photodynamic therapy intervention protocol. **(A)** Prospective enrollment of patients into the experimental group based on predefined inclusion/exclusion criteria. **(B)** Preoperative (4 hours before surgery) oral administration of 5-aminolevulinic acid (5-ALA, Alasens^®^, 20 mg/kg body weight). **(C)** Intraoperative visualization and grading of protoporphyrin IX (PpIX) fluorescence using an operating microscope (Leica M720 OH5, Carl Zeiss, Germany) equipped with a blue-violet light source (400 nm) to assign a Visual Fluorescence Score (FIS: 1=weak, 2=moderate, 3=intense). **(D)** Quantitative measurement of PpIX accumulation in the tumor using a laser biospectroscopy system (LESA-01-BIOSPEC, Biospec, Moscow, Russia) to calculate the fluorescence index (FI, arbitrary units). **(E)** Maximal safe tumor resection guided by real-time visual fluorescence and biospectroscopic feedback. (E1) Post-resection mapping of the fluorescence index in the resection cavity, specifically targeting the tumor matrix (in skull base tumor) and the peritumoral zone with suspected invasion of the brain arachnoid membrane. **(F)** Biopsy collection from the tumor matrix and peritumoral zone for histopathological and immunohistochemical analysis. **(G)** Photodynamic therapy using a diode laser (LFT-02-BIOSPEC, 635 nm, Biospec, Moscow, Russia) applied to the tumor bed in 2-5-minute fractions. Cycles of PDT **(G)** were interrupted with repeated biospectroscopic assessments (E1), with continuous thermographic monitoring. The procedure was terminated upon achieving target PpIX photobleaching, indicated by an FI reduction to baseline levels of normal tissue. **(H)** Final post-intervention biopsy acquisition from the matrix and peritumoral zones for subsequent analysis of PDT-induced cytotoxic effects via histology and immunofluorescence microscopy.

For patients in the experimental group (n=23), the following integrated protocol of fluorescence-guided surgery and photodynamic therapy was performed (**Figure 1**). The integrated protocol of fluorescence-guided surgery and photodynamic therapy used for the experimental group is protected by Russian Federation Patent No. 2840600 (“Method for preventing recurrence of intracranial meningiomas after surgical treatment” application No. 2024117754, entered into the State Register of Inventions on 26 May 2025).

#### 2.2.1 Preoperative preparation and 5-ALA administration

Four hours before surgery, patients received a freshly prepared solution of 5-aminolevulinic acid (5-ALA, Alasens^®^) at a dose of 20 mg/kg body weight (**Figures 1A, B**). The powder was dissolved in 50 mL of drinking water and administered orally under supervision. Patients were subsequently protected from direct sunlight and bright indoor light to prevent skin photosensitivity.

#### 2.2.2 Fluorescence-guided tumor resection

**Figure 2:**
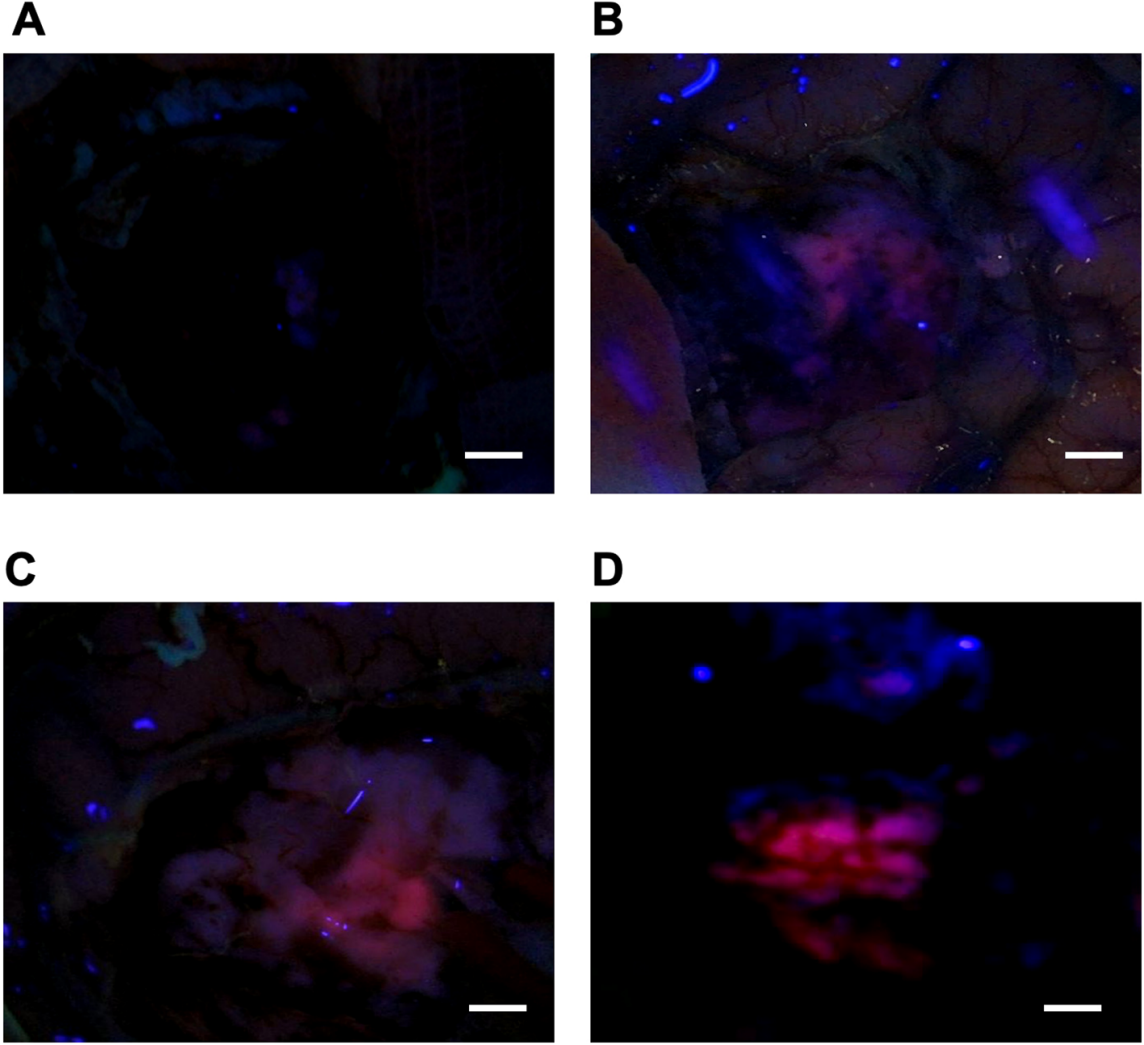
Representative intraoperative photographs demonstrating the grading scale for visible 5-ALA-induced fluorescence. **(A)** There is no pink glow. **(B)** Weak fluorescence (FIS 1): a faint pink glow. **(C)** Moderate fluorescence (FIS 2): a distinct pink fluorescence is observed. **(D)** Intense fluorescence (FIS 3): brilliant, vivid pink fluorescence illuminating the surgical field. All images were acquired under blue-violet light (400 nm) using an operating microscope. Scale bar = 5 mm. FIS – Fluorescence Intensity Score.

Following standard craniotomy and dural opening, the tumor was visualized using an operating microscope (Leica M720 OH5, Carl Zeiss, Germany) equipped with a dedicated blue-violet light source (wavelength 400 nm) for fluorescence detection (**Figure 1C**). The intensity of visible protoporphyrin IX (PpIX) fluorescence in the tumor mass and peritumoral areas was assessed and documented using photo and video recording. Fluorescence Intensity Score (FIS) was graded according to a 4-point scale: FIS 0 - no visible fluorescence, FIS 1 - weak; FIS 2 - moderate; FIS 3 – intense (**Figure 2**).

Simultaneously, quantitative measurements of PpIX accumulation were performed using the laser electronic-spectral system LESА-01-BIOSPEC (BIOSPEC LLC, Moscow, Russia). The fluorescence index (FI) was automatically calculated by the device’s proprietary software as the ratio of the PpIX fluorescence intensity to the intensity of the back-reflected laser signal (**Figure 1D**), expressed in arbitrary units (a.u.). Measurements were taken the tumor core tissue, and from the tumor matrix and the adjacent brain parenchyma after tumor resection.

Tumor resection was then performed with the goal of achieving a maximal safe resection, guided by both the real-time visual fluorescence and the quantitative feedback from the biospectroscopy system (**Figures 1E, E1**). The entirety of the resected tumor tissue was collected and submitted for standard histopathological examination to confirm the diagnosis and WHO grade.

#### 2.2.3 Intraoperative photodynamic therapy

After the completion of the main resection phase, PDT was administered to the surgical cavity (**Figure 1G**).

The fluorescence index was re-measured at multiple points within the resection cavity, including the tumor matrix and the peritumoral zone. Additional biopsy specimens were taken from the peritumoral zone and tumor matrix (2–3 samples from each zone, sample volume 10–30 mm³) for subsequent histopathological, immunohistochemical and immunofluorescence analysis.

The resection cavity was irradiated using a diode laser LFT-02-BIOSPEC system (BIOSPEC LLC, Moscow, Russia) with a power output of 2.0 W and a wavelength of 635 nm. The laser light was delivered via a sterile end-face optical fiber. PDT was administered in short, 2-minute and 5-minute sessions. After each session, biospectroscopic measurements and visual fluorescence assessments were repeated. Throughout the irradiation, tissue temperature in the treatment field was continuously monitored using the TermoLabs thermographic system (LETI, Saint Peterburg). To prevent thermal damage, the PDT session was immediately paused if the tissue temperature exceeded 36.9 °C and was resumed only after it cooled down to 36 °C. All operating room personnel used protective goggles with filters blocking the 635 nm wavelength. The termination of the photodynamic therapy procedure was determined by spectroscopic monitoring of the fluorescence index (FI) in the target area. The PDT session was concluded once the FI in the zone of interest decreased to values corresponding to the background fluorescence recorded from the intact dura mater. The intact dura mater served as an internal biological baseline for each specific patient. This approach ensured that the irradiation was continued until the photosensitizer-induced fluorescence was effectively quenched to the level of non-tumorous background tissues.

Following the final PDT session, the following dosimetric parameters were calculated using the formula:


$$Light\;Dose\;\left(J/cm^{2}\right):\frac{P\times T}{S}$$

, where *P* is the laser output power (Watts), *T* is the total irradiation time (seconds), and *S* is the irradiated area (cm²).

Power Density (mW/cm^2^) is the photon delivery unit expressed by milliwatt per square centimeter.

Final tissue samples were collected from the peritumoral zone and tumor matrix (2–3 samples from each zone, sample volume 10–30 mm³) for detailed pathomorphological examination to assess the cytotoxic effects of the PDT. Paired biopsy pre-PDT and post-PDT specimens were consistently obtained from identical anatomical locations (**Figures 1F, H**).

The postoperative protocol included standardized neurological assessments (on days 1, 3, and 7) and early neuroimaging (MRI or CT) within the first 24 hours for all study participants.

### 2.3 Histopathological and immunohistochemical analysis

Tissue specimens for histopathological examination were collected from three distinct sources: the main resected tumor mass, for definitive histopathological diagnosis and WHO grading; targeted biopsy specimens from the tumor matrix and the peritumoral zone, obtained intraoperatively from each patient in the experimental group at two distinct time points: prior to the initiation of photodynamic therapy and immediately following the completion of the final photodynamic therapy and biospectroscopy session. All collected specimens were fixed in 10% neutral buffered formalin, subsequently dehydrated through a standardized graded ethanol series, cleared in xylene, and embedded in paraffin blocks.

For general morphological assessment, serial sections (4-5 µm thick) were stained with hematoxylin and eosin (H&E). For the definitive diagnosis and grading of the main tumor mass according to the 5th edition of the WHO Classification of CNS Tumors (2021), a comprehensive immunohistochemical panel was performed. The following primary antibodies from Dako (Agilent, Denmark) were used: anti-SSTR2(Somatostatin Receptor 2), clone UMB1, RTU; anti-EMA (Epithelial Membrane Antigen), clone E29, RTU; anti-EGFR (Epidermal Growth Factor Receptor), clone H11, RTU; anti-VEGF (Vascular Endothelial Growth Factor), clone VG1, dilution 1:100; anti-PR (Progesterone Receptor), clone PgR 636, RTU; anti-CD3 (T-cell marker), clone F7.2.38, RTU; anti-CD8, (Cytotoxic T-cell marker), clone C8/144B, RTU; anti-Ki-67 (Proliferation marker), clone MIB-1, dilution 1:100.

Consecutive sections from the pre- and post-PDT biopsy samples (tumor matrix and peritumoral zone) were used to assess the specific effects of therapy. The following antibody were evaluated: anti-PR (Clone PgR 636, RTU, Dako) was assessed in the tumor matrix, anti-Caspase-3 (apoptotic activity, clone C92-605, dilution 1:200, BD Biosciences, USA) was quantified in the peritumoral zone.

For all IHC procedures, antigen retrieval was performed using the EnVision FLEX Target Retrieval Solution (high or low pH, as required for each antibody). The EnVision FLEX/HRP visualization system (Dako, Denmark) was used for detection according to the manufacturer’s protocol, with 3,3’-Diaminobenzidine as the chromogen (Sigma-Aldrich, Darmstadt, Germany). All stained sections were examined and digitally captured using a Leica DM2500 M research microscope equipped with a DFC320 camera and the IM50 image manager software (Leica Microsystems, Wetzlar, Germany). The proliferative index (Ki-67) and PR expression were assessed as the percentage of positively stained nuclei among the total number of tumor cells counted in at least ten representative high-power fields (HPF, 400x magnification) in the tumor matrix. Apoptotic activity (Caspase-3) was quantified as the number of positively stained cells per mm² of tissue in the peritumoral zone. The expression patterns and intensity of SSTR2, EMA, EGFR, VEGF, CD3, and CD8 were described semi-quantitatively. Quantitative analysis was performed using ImageJ software (National Institutes of Health, USA). For each patient, the pre- and post-PDT samples from matched anatomical zones (tumor matrix and peritumoral zone) were compared. Data are presented as the mean value ± standard deviation.

### 2.4 Immunofluorescence microscopy

Inverted confocal microscopy was performed on cryosections sections from the tumor matrix samples obtained before and after photodynamic therapy. Tissue sections were stained with the following fluorescent probes to assess various cellular components: DAPI (4’,6-diamidino-2-phenylindole, Ibidi, Gräfelfing, Germany) to visualize cell nuclei; TMRM (Tetramethylrhodamine Methyl Ester, 1 μmol/L, Thermo Fisher Scientific, Waltham, Massachusetts, USA) to detect active mitochondria and assess mitochondrial membrane potential; Anti-Hsp70 Antibody (SPA810, Stress-Marq Biosciences Inc, Victoria, Canada) to visualize the expression and localization of heat shock protein 70. A secondary antibody conjugated to a fluorophore (Alexa Fluor 488) was used for detection. The samples were then washed and placed in a thin-bottomed Ibidi μ-Dish 35-mm (80136, Ibidi, Gräfelfing, Germany) and covered with a cover glass. The material was visualized by Leica TCS SP8 (Leica Microsystems, Wetzlar, Germany) fitted with argon and helium-neon lasers. The following laser lines and detection windows were used for sequential image acquisition to minimize crosstalk: DAPI (excitation at 405 nm; emission detection at 415–480 nm); Hsp70 (via secondary antibody, e.g., Alexa Fluor 488, excitation at 488 nm; emission detection at 495–560 nm); TMRM (excitation at 561 nm; emission detection at 570–630 nm); protoporphyrin IX (excitation at 405 nm; emission detection at 670–750 nm). An HC PL APO 63x/1.40 OIL CS2 oil immersion lens was used. Images were obtained at a resolution of 1024 by 1024 pixels with an average of three along each scanning line. Throughout the experiment, the same system settings were used. Images of unstained samples were used as a control for autofluorescence. At least 10 images were obtained from each sample.

### 2.5 Statistical analysis

Statistical analysis was performed using GraphPad Prism version 10.0.0 (GraphPad Software, USA). The normality of data distribution for continuous variables was assessed using the Kolmogorov-Smirnov and Shapiro-Wilk tests. Based on the distribution and type of data, the following specific tests were applied to address the study’s aims: to evaluate the association between the visible fluorescence level (ordinal qualitative variable: FIS 0-3) and various factors or preoperative treatment history (nominal qualitative variables), the Chi-square test or Fisher’s exact test was used, as appropriate. For comparisons of the biospectroscopy fluorescence index (continuous variable) across the different grades of visible fluorescence level (ordinal qualitative variable), the Kruskal-Wallis test with Dunn’s *post-hoc* test for multiple comparisons was employed. To compare the proportion of patients with different extent of tumor resection between the control and experimental groups, Fisher’s exact test was used due to the relatively small sample sizes in the groups. For comparisons of continuous variables (age, fluorescence index, caspase-3 expression) between two independent groups (pre-PDT vs. post-PDT samples within the experimental group), the Mann-Whitney U test was used. We used the mean **±** standard deviations for normally distributed continuous data, median [IQR] for non-normally distributed continuous data and percentages for categorical variables. A p-value of less than 0.05 was considered statistically significant. All graphs and figures were generated using GraphPad Prism version 10.0.0.

## 3 Results

### 3.1 Patient characteristics

A total of 58 patients with recurrent intracranial meningiomas were included in the study into two groups: the study group (FGS+PDT, n=23) and the control group (standard microsurgery, n=35). The baseline demographic and clinical characteristics of both groups are summarized in **Table 1**. The groups were well-matched for key prognostic factors: age, sex, WHO grade at initial diagnosis and at recurrence, the number of prior interventions.

### 3.2 5-ALA-induced fluorescence in recurrent meningiomas: intensity, quantitative assessment, and impact on extent of resection

**Figure 3:**
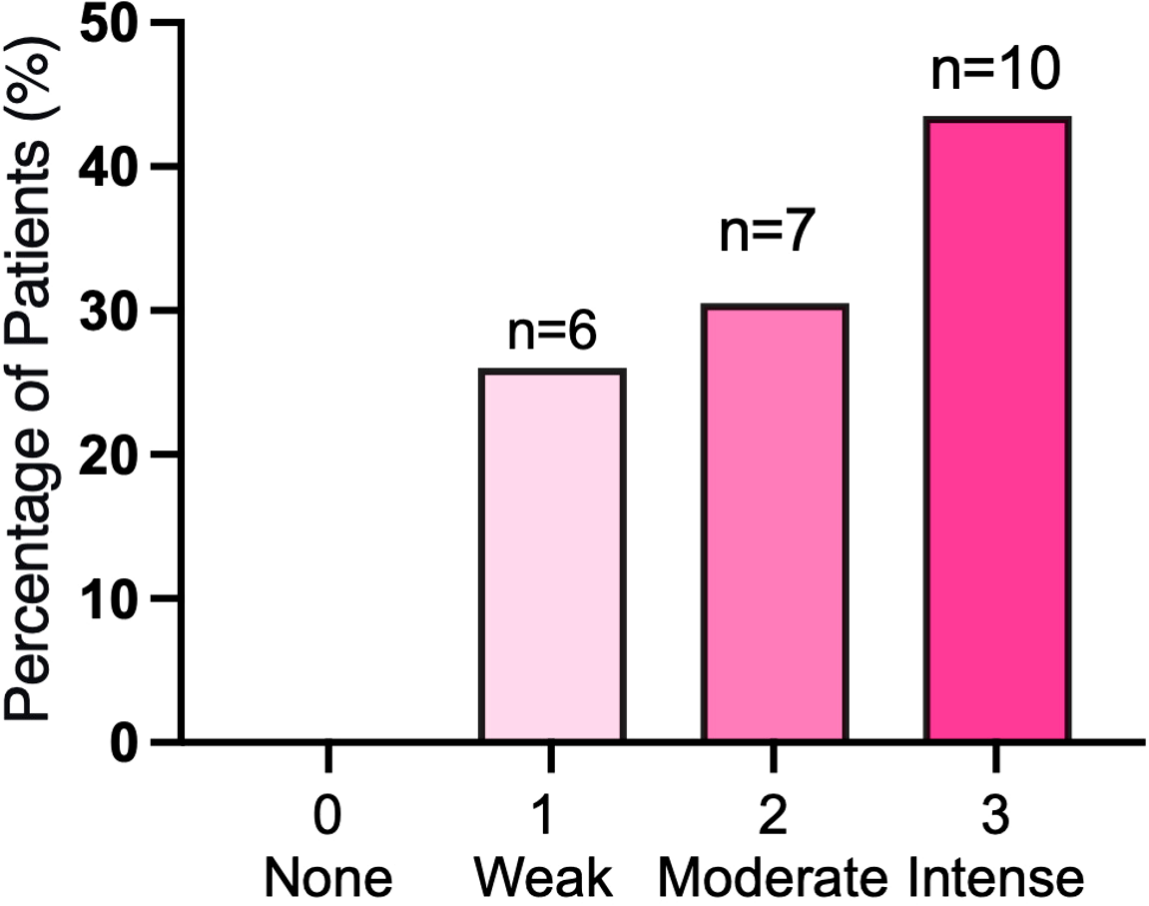
Distribution of 5-ALA-induced fluorescence intensity in the experimental group (n=23). Bar chart illustrating the prevalence of different levels of visible protoporphyrin IX fluorescence following 5-ALA administration in the experimental cohort (n=23). Fluorescence Intensity Score (FIS) was defined as: 1 = weak, 2 = moderate, 3 = intense. No tumors scored FIS 0.

The accumulation of protoporphyrin IX (PpIX) following 5-ALA administration was assessed intraoperatively in 23 patients of the experimental group (FGS+PDT). The distribution of visible fluorescence intensity within this cohort is summarized in **Figure 3**.

The majority of recurrent meningiomas in this group exhibited intense fluorescence, with 43,5% (n=10/23) classified as Fluorescence Intensity Score (FIS) 3. FIS 2 was observed in 30,5% (n=7/23) of cases, and weak fluorescence (FIS 1) was present in 26,0% (n=6/23). A complete absence of visible fluorescence (FIS 0) was not observed in any of the patients who received 5-ALA.

Quantitative assessment using intraoperative biospectroscopy was performed on the tumor surface prior to resection. The median fluorescence index (FI) in the tumor core was 30.0 a.u. [20.0 - 41.0]. The quantitative PpIX accumulation the three FIS groups were not statistically distinct. The median FI for tumors with weak visual fluorescence (FIS 1) was 25 a.u. [12.0 – 31.25], for FIS 2–22 a.u. [20.0 – 50.0]), and for FIS 3–31 a.u. [43.0 – 25.0]. The Kruskal-Wallis test found no statistically significant difference in the FI values among the three FIS groups (p = 0.38).

**Figure 4:**
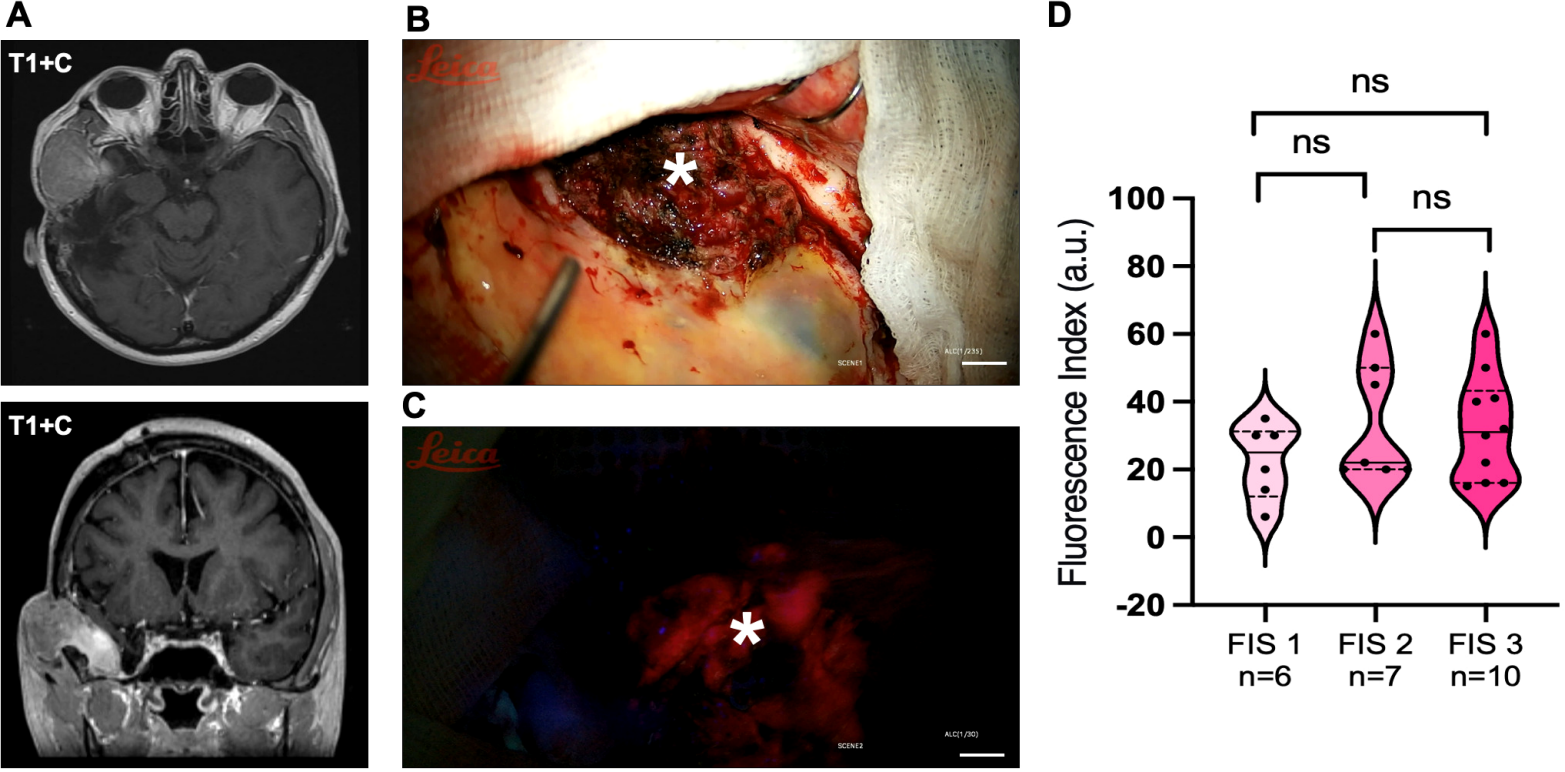
Relationship between visual and quantitative fluorescence assessment. **(A)** Representative preoperative magnetic resonance image of a recurrent atypical meningioma. Axial and coronal T1-weighted contrast-enhanced image demonstrates a large, homogeneously enhancing extra-axial mass (75 x 47 x 57 mm) in the right middle cranial fossa with significant extracranial extension. Recurrent atypical meningioma was diagnosed 36 months after initial surgical resection. **(B)** Representative intraoperative photograph of a recurrent meningioma (white asterisk) under white light. Scale bar = 5 mm. **(C)** The same surgical field under blue-violet light (400 nm) demonstrating intense fluorescence (FIS 3). Scale bar = 5 mm. **(D)** Violin plot illustrating the distribution of the quantitative fluorescence index (FI, arbitrary units) across the three Visual Fluorescence Score (FIS) groups. The solid line within each violin represents the median, and the dashed lines represent the interquartile range, ns – non-specific, n=23.

As illustrated in **Figure 4**, the distribution of FI values demonstrated extensive overlap between all three visual score categories. Numerous individual tumors with a visual grade of FIS 1 (weak) exhibited FI values that fell within the range of FIS 3 (intense) tumors, and vice versa.

Given the observed variability in visible fluorescence intensity, we investigated its association with key patient and tumor characteristics. A comprehensive analysis was conducted to evaluate the relationship between the Visual Fluorescence Score and factors including tumor location (skull base vs. non-skull base), preoperative MRI features (e.g., presence of peritumoral edema), tumor biology (WHO grade at recurrence, index Ki67, endothelial cell proliferation, lymphocyte infiltration, VEGF expression), and history of prior adjuvant therapy (radiotherapy). The analysis revealed no statistically significant associations between the intensity of visible fluorescence and any of the clinical, radiological, or histopathological factors assessed (p > 0.05 for all comparisons, Chi-square or Fisher’s exact test).

To assess the potential benefit of fluorescence guidance on surgical radicality, we compared the extent of resection between the study and control groups. For this analysis, the extent of resection tumor was dichotomized into high radicality (Simpson Grades I-II) and low radicality (Simpson Grades III-V). A gross-total resection (Simpson I-II) was achieved in 95.6% (22 of 23) of patients in the fluorescence-guided surgery group. In contrast, in the control group undergoing conventional microsurgery, a similar degree of radicality was achieved in 77.1% (27 of 35) of patients. However, this difference did not reach statistical significance, showing only a trend toward improvement (p = 0.07, Fisher’s exact test).

### 3.3 Feasibility and delivery of photodynamic therapy for recurrent meningiomas

Following maximal safe resection under fluorescence and biospectroscopy guidance, the feasibility of subsequent photodynamic therapy was evaluated. PDT was successfully delivered and well-tolerated by all 23 patients in the experimental group. Quantitative biospectroscopy was performed in the resection cavity, specifically targeting the peritumoral zone with suspected arachnoidal invasion and the tumor matrix in skull base meningiomas.

**Figure 5:**
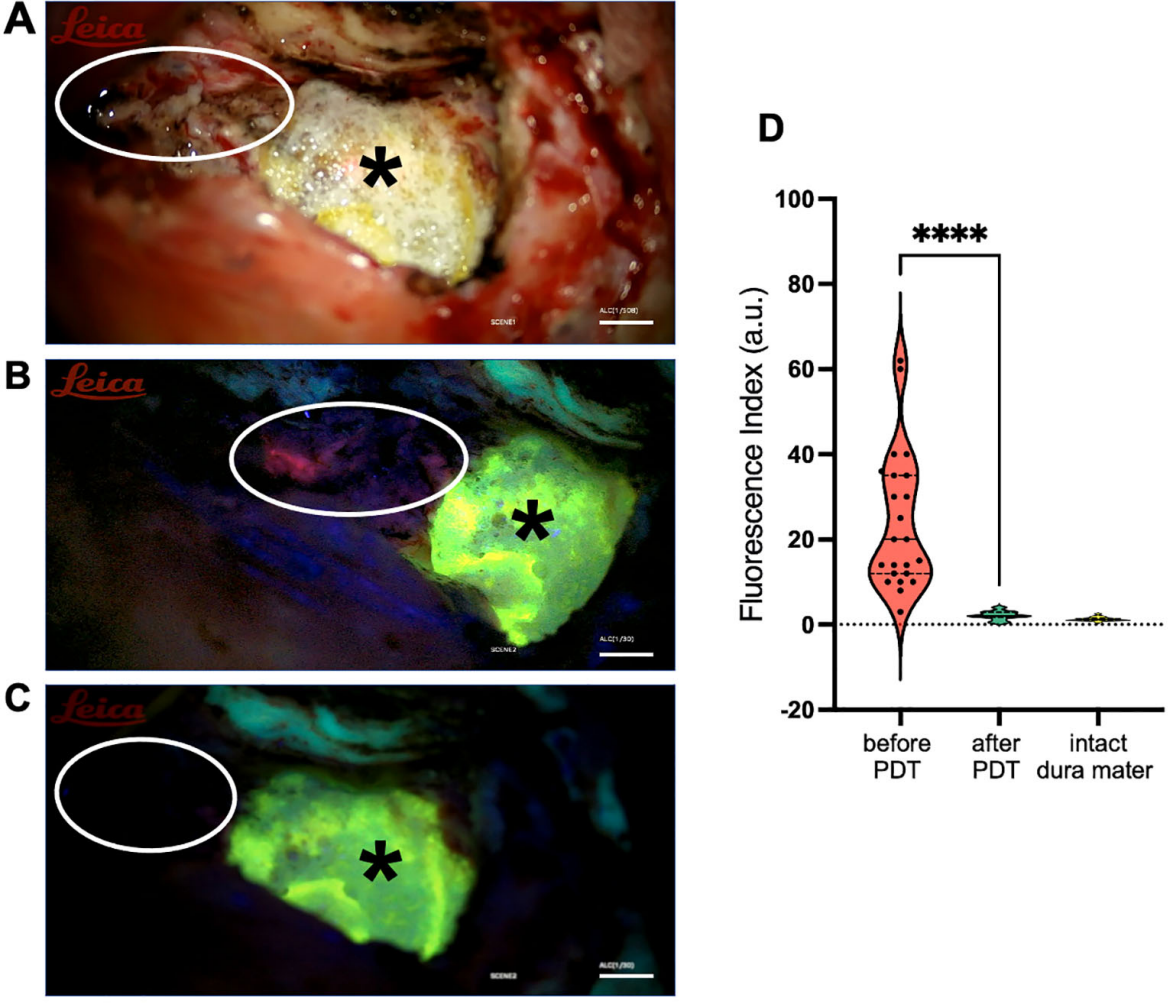
Intraoperative photodynamic therapy. **(A)** The resection bed under white light after atypical meningioma of middle cranial fossa removal. A sheet of TachoSil^®^ surgical patch has been applied to the temporal pole for hemostasis. Scale bar = 5 mm. **(B)** The same field under blue-violet light (400 nm) prior to photodynamic therapy (PDT), demonstrating residual PpIX fluorescence in the tumor matrix and peritumoral zone (white oval). The patch of TachoSil^®^ exhibits intense autofluorescence (green signal, black asterisk). Scale bar = 5 mm. **(C)** The resection bed under blue-violet light after completed PDT, showing visible photobleaching (white oval, quenching of the pink fluorescence). Scale bar = 5 mm. **(D)** Violin plot showing the distribution of fluorescence index (FI) values in the target zone before and after PDT, with reference values from control tissues. Solid line represents median; dashed lines represent IQR. ****p< 0.0001, Mann-Whitney U test, n=23.

The baseline fluorescence index (FI) in the resection bed prior to PDT was substantial, with a median of 20.0 a.u. [12.0 – 35.0], confirming the presence of residual protoporphyrin IX (PpIX) as a target for therapy. This value was significantly higher than the FI recorded in intact dura mater: 1.2 a.u. [1.0 – 1.5]. The median area of the irradiated surgical bed was 7 cm² [2.4 – 29.0]. The median total illumination time required to achieve the endpoint of PpIX photobleaching, defined as a reduction in FI to levels recorded in normal, non-neoplastic brain tissue, was 12.0 minutes [7 - 18]. Post-PDT biospectroscopy confirmed a significant reduction in the FI within the treated area to a median of 2.0 a.u. [1.7 – 2.9], indicating successful activation of the photosensitizer (**Figure 5**). The dosimetric parameters calculated for the procedure were as follows: the median Light Dose delivered was 257 J/cm² [66 – 617], with a median Power Density of 429 mW/cm² [66 – 858].

No adverse events related to 5-ALA or PDT were observed in any of the 23 patients in the experimental group. Specifically, there were no instances of phototoxic skin reactions, neurological deterioration, or other complications linked to the experimental intervention. The Karnofsky Performance Status (KPS) score of the patients remained stable throughout the study period: 16 patients (69.6%) had a KPS score of 70–80%, while 7 patients (30.4%) scored 90–100%. According to postoperative MRI or CT scans no cases of symptomatic or pathological exacerbation of peritumoral oedema requiring prolonged corticosteroid therapy or surgical re-intervention were recorded. The volumetric changes in the oedema zone were consistent with standard postoperative expectations following microsurgical resection of high-grade meningiomas.

Patients in the experimental group were followed for a median of 16 months [IQR 11.0 – 22.0] postoperatively. No local tumor recurrence or progression was observed in any patient during this follow-up period.

### 3.4 Histopathological and immunofluorescence analysis of photodynamic therapy effects in recurrent meningiomas

To characterize the biological effects of photodynamic therapy at the cellular level, a comparative histopathological and immunohistochemical analysis was performed on paired biopsy samples from the tumor matrix and peritumoral brain obtained before and immediately after PDT completion.

**Figure 6:**
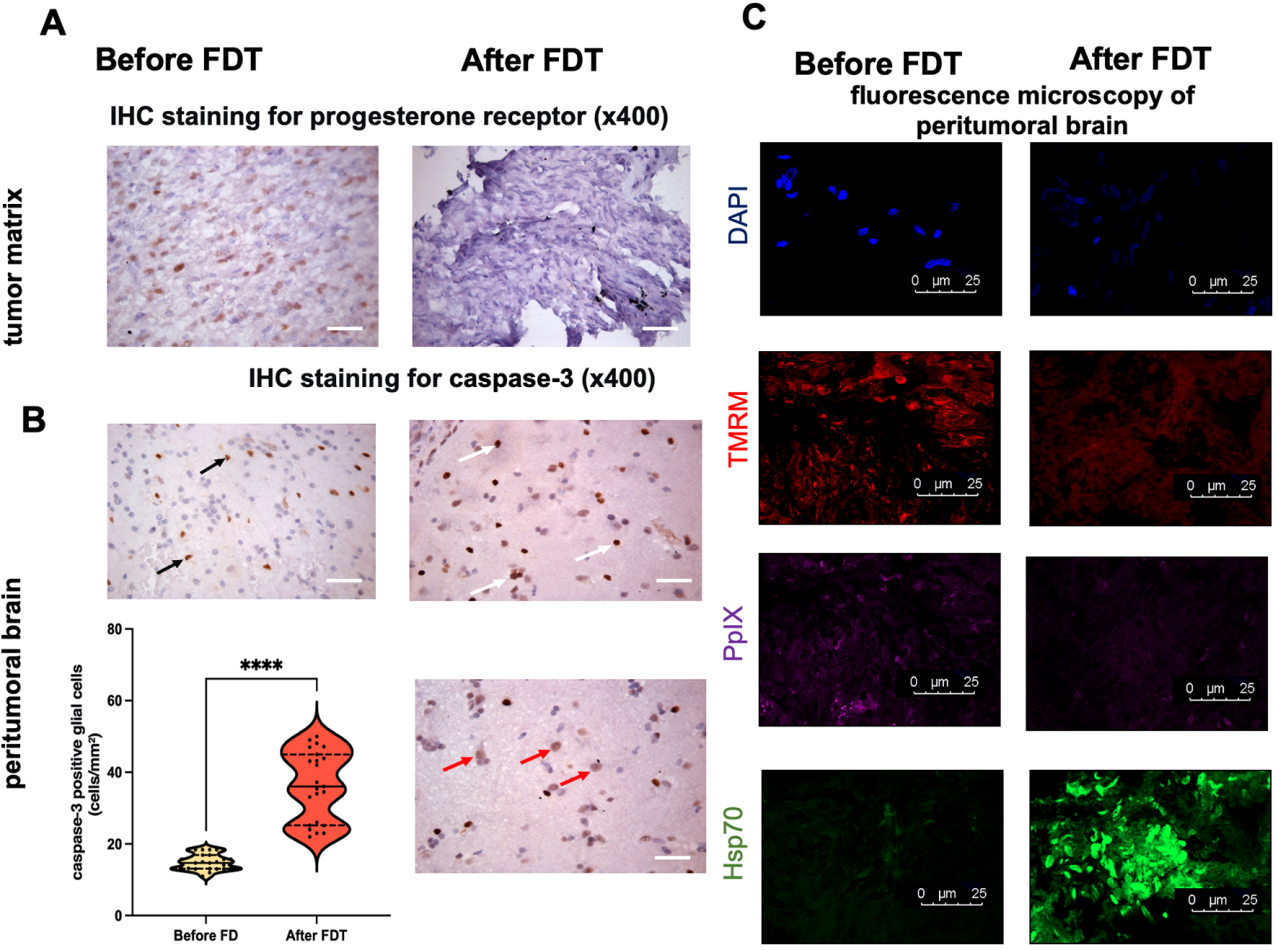
Histopathological and immunofluorescence analysis of biopsy samples before and after photodynamic therapy **(A)** Representative immunohistochemical staining of atypical meningioma matrix before PDT, showing strong positive nuclear staining for progesterone receptors (PR) in tumor cells. A matched sample from the same zone after PDT, demonstrating complete loss of nuclear PR immunoreactivity. *Scale bar: 300 µm.***(B)** Representative image of samples from the peritumoral zone before PDT, showing low baseline expression of caspase-3 in glial cells (black arrow). Matched peritumoral zone after PDT, demonstrating markedly increased caspase-3 expression in glial cells (white arrow) and the novel appearance of caspase-3 positivity in neuronal nuclei (red arrow), indicative of apoptosis. *Scale bar: 300 µm.* Violin plot quantifying the density of caspase-3 positive glial cells (cells/mm²) before and after PDT. Solid line represents mean; dashed lines represent ±SD ****p< 0.0001, Mann-Whitney U test, n=46. **(C)** Representative images of fluorescence microscopy of samples from the peritumoral zone before PDT and matched samples from the same region after PDT. DAPI staining - blue, TMRM staining – red, PpIX fluorescence – magenta, Hsp70 - green. *Scale bar: 25 µm.*.

In pre-PDT samples from tumor matrix, tumor cells exhibited high nuclear PR expression, with a mean density of 79.7 ± 10.3 positive cells per mm² (**Figure 6A**). In contrast, post-PDT samples from the same anatomical site demonstrated a complete absence of nuclear PR immunoreactivity (**Figure 6A**).

The cytotoxic effect of photodynamic therapy was further evaluated by assessing the expression of the pro-apoptotic marker caspase-3 in the peritumoral brain tissue before and after treatment. Prior to PDT, a low baseline level of apoptosis was observed, with caspase-3 expression localized exclusively to glial cells at a density of 14.8 ± 2.2 positive cells per mm² (**Figure 6B**). Following PDT, a significant and substantial increase in apoptotic activity was documented. The density of caspase-3-positive glial cells increased to 36.3 ± 9.6 cells per mm² (p< 0.0001) (**Figure 6B**). Furthermore, a novel post-PDT finding was the appearance of caspase-3 immunoreactivity within neuronal nuclei, with a density of 2.8 ± 1.5 positive cells per mm² (**Figure 6B**).

To delineate the subcellular effects of photodynamic therapy, we performed confocal microscopy analysis on peritumoral zone samples that exhibited elevated baseline fluorescence index and were subsequently targeted by PDT (**Figure 6C**). Comparative analysis before and after treatment revealed a consistent pattern of profound cellular damage. After-PDT samples demonstrated a marked reduction in cellularity, as evidenced by decreased DAPI staining, indicating nuclear degradation and loss of cellular integrity. Concurrently, a sharp decline in TMRM fluorescence signal was observed, confirming the collapse of mitochondrial membrane potential and the irreversible commitment to apoptosis. The near-complete degradation of protoporphyrin IX (PpIX) fluorescence validated the successful achievement of target photobleaching during the procedure. Crucially, we documented a significant overexpression of Hsp70, a canonical marker of severe proteotoxic and oxidative stress, illustrating the direct protein-damaging effects of PDT-generated ROS.

## 4 Discussion

In this single-center, prospective cohort study, we evaluated the feasibility of a novel integrated approach combining 5-aminolevulinic acid fluorescence-guided surgery with intraoperative photodynamic therapy for recurrent atypical and anaplastic intracranial meningiomas. The management of these recurrent lesions remains a formidable neurosurgical challenge, as they often exhibit aggressive local growth and are difficult to further interventions after exhausting standard treatments of surgery and radiotherapy (4–6). Our findings demonstrate that this combined strategy is not only feasible and safe but also provides improvements in surgical radicality and elicits profound biological effects at the cellular level.

A cornerstone finding of our investigation is the consistent and universal accumulation of 5-ALA-induced protoporphyrin IX in recurrent atypical and anaplastic meningiomas. Visible fluorescence was observed in 100% of our prospective cohort, a result that strongly validates the applicability of fluorescence-guided surgery for this specific pathology. This finding gains particular significance in the context of the existing literature. A review by Dijkstra et al. (25) reported high but variable sensitivity (92–98%) and specificity (95%) for 5-ALA across studies, predominantly involving mixed cohorts of primary and recurrent meningiomas, the majority being WHO grade 1 (25). Similarly, a large prospective study by Potapov et al. (26), which included 101 patients with meningiomas (WHO grades 1-3), contained only 9% recurrent cases (26). Consistent with our results, these researches did not found correlation between fluorescence intensity and WHO grade or histological subtype, noting frequent heterogeneity in fluorescence patterns. Large-scale studies focusing exclusively on recurrent meningiomas are absent from the literature, with evidence limited primarily to individual case reports (27–29). Consequently, our results from a homogeneous cohort of 23 recurrent tumors provide unique evidence that the process of recurrence itself, potentially associated with altered cellular metabolism and disruption of the blood-brain barrier, may facilitate consistent and reliable PpIX synthesis. This high rate of PpIX accumulation is a critical enabler, allowing neurosurgeons to leverage a well-established tool from glioma surgery (30) for this new clinical scenario.

The utility of this fluorescence was directly translated into a significantly higher rate of gross-total resection (Simpson Grades I-II) in the FGS group compared to controls group (95.6% vs. 77.1%). However, this observation showed only a borderline statistical trend (p = 0.07). While not reaching the conventional 0.05 threshold, this finding is clinically encouraging and likely reflects the added value of intraoperative fluorescence in the challenging surgical landscape of recurrent disease. It is probable that the relatively small cohort size in this feasibility trial limited the statistical power to definitively confirm this improvement. Future larger-scale studies are necessary to determine if this trend translates into a statistically significant clinical advantage.

Recurrent meningiomas are often embedded in post-operative scar tissue, making the discrimination between tumor and adhered brain parenchyma or fibrotic planes exceptionally difficult under conventional white light (9, 10). Beyond our study, which specifically compared the extent of meningioma resection using 5-ALA with a control group, similar controlled investigations are absent from the literature. In a retrospective study by Cornelius et al. (31), which included 31 patients with primary WHO grade I (61%) meningiomas, the improvement in the radicality of resection was assessed subjectively by the operating surgeon without comparison to a control group of patients (31). This underscores the methodological uniqueness of our work. Our experience confirms that 5-ALA fluorescence effectively highlights superficial infiltrative nests and residual tumor tissue within these adhesions, thereby improving the surgeon’s ability to achieve a more radical and anatomically precise resection, which is a key prognostic factor for delaying further recurrence.

The most significant methodological insight from our study is the demonstrated discordance between subjective visual fluorescence assessment and objective, quantitative measurement of protoporphyrin IX (PpIX). We found no statistically significant correlation between the Visual Fluorescence Score (FIS) and the quantitative Fluorescence Index (FI) obtained via biospectroscopy. Heterogeneity in visible fluorescence has also been reported in the works of Dijkstra et al. (25) and Potapov et al. (26) (25, 26). In a study by Scheichel et al. (2019), which included 11 patients, fluorescence was observed in 100% of meningiomas with bone and soft tissue infiltration (32). However, the intensity of visible fluorescence was not uniform—strong and homogeneous fluorescence was noted in 81.8% of cases, while vague and heterogeneous fluorescence was present in 18.2%. This reinforces the conclusion that the human eye is a poor judge of the actual PpIX concentration in tissue. This subjectivity can be attributed to several factors, including variable background illumination, the surgeon’s individual perception, and optical properties of the tissue, such as blood content and the thickness of the overlying meninges. These findings underscore the necessity of employing more objective methods for quantifying PpIX accumulation in meningioma tissue. Supporting this notion, a study by Knipps et al. (33) involving 13 patients with primary meningiomas demonstrated that a spectrometer exhibited high sensitivity in detecting meningioma cells, whereas the surgical microscope showed false negative results and missed residual tumor cells in more than one half of the cases (33). This finding has profound implications, especially for PDT.

Furthermore, the absence of a significant association between fluorescence intensity and various clinical-pathological factors such as WHO grade, tumor location, or prior treatment history is, paradoxically, a positive clinical outcome. It suggests that the ability to accumulate 5-ALA is a ubiquitous property of recurrent meningiomas, independent of their specific subtype or history. This universality greatly enhances the clinical applicability of the technique, as neurosurgeons can anticipate visible fluorescence and plan for FGS and subsequent PDT without the need for complex pre-operative patient selection protocols.

A critical distinction of our PDT protocol lies in its personalized dosimetric approach. While the majority of the clinical literature on 5-ALA PDT in neurosurgery is dedicated to glioblastomas—and in contrast to those studies which have typically employed fixed light doses, such as the 200 J/cm² used in the work by Vermandel et al. (2021) (34), we utilized fluorescence index measurements to adapt the therapy. Our data strongly advocate for the integration of quantitative biospectroscopy as an indispensable tool to guide PDT dosimetry. By providing a real-time, objective measure of PpIX accumulation, biospectroscopy ensures that light delivery is tailored to the actual target concentration, thereby maximizing the potential for a potent cytotoxic effect while establishing a standardized endpoint for therapy (photobleaching). This methodology represents a significant paradigm shift from a one-size-fits-all dose to a biologically guided, patient-specific treatment. The significant post-PDT reduction in FI, achieving levels comparable to normal brain tissue, confirms the biological activity of the treatment and the successful achievement of target photobleaching. This objective endpoint is a major advancement over subjective visual assessment and is supported by *in vitro* evidence that PpIX concentration directly correlates with PDT efficacy in meningioma cells (20, 21, 35). We believe this methodology is highly translatable to other biospectroscopy platforms. By using an in-patient internal control (normalization to the intact dura mater) rather than an absolute numerical threshold, the protocol accounts for inter-device variability and differences in light-tissue interaction. Other platforms can implement a similar relative thresholding strategy by calibrating their signal-to-background ratio against healthy intracranial structures, which remains a robust and reproducible approach across different fluorescence detection systems.

The clinical feasibility and safety of our protocol of combination of FGS and FDT is objectively reflected in the stability of the Karnofsky Performance Status scores. The fact that all 23 patients maintained a KPS score of 70% or higher postoperatively demonstrates that the PDT-induced apoptotic changes did not compromise the patients’ functional independence. This preservation of high-level neurological function is a key safety indicator, especially given the aggressive nature of recurrent high-grade meningiomas and the targeted application of PDT at the infiltrative margins.

At a median follow-up of 16 months, no cases of local recurrence were observed in the experimental group. However, this finding should be interpreted strictly as a preliminary observation rather than definitive proof of long-term local control. Given the aggressive biological behavior and high recurrence rates inherent to atypical and anaplastic meningiomas, the current follow-up duration is insufficient to draw final conclusions regarding the long-term oncological efficacy of the integrated PDT protocol. Continuous long-term surveillance of this cohort is essential to evaluate the durability of the observed clinical response. Also Takahashi et al. (36) reported that prior radiotherapy could limit the effectiveness of PDT with talaporfin sodium (36), our results with 5-ALA in a population where many had received prior radiotherapy (39.1%) suggest a potent cytotoxic effect.

A central question remains whether the observed clinical benefits stem from the improved visualization provided by FGS or the direct cytotoxic effects of PDT. Our findings suggest that these two modalities are inherently complementary and synergistic. Notably, even after achieving macroscopically total resection, intraoperative biospectroscopy revealed a high fluorescence index (FI) in the resection bed (median 20.0 a.u. vs. 1.2 a.u. in intact dura), confirming the presence of residual PpIX-accumulating tumor cells. These “hotspots” of microscopic infiltration, particularly within the tumor matrix of skull base lesions or the peritumoral zone, represent the primary targets for PDT. By inducing apoptosis in these spectroscopically identified but surgically inaccessible residuals, PDT provides an additional layer of local control that exceeds the limits of standard microsurgical radicality. This combinatorial strategy is further supported by *in vitro* evidence indicating that the susceptibility of meningioma cells to 5-ALA-PDT is highly variable and dependent on intrinsic factors, such as ferrochelatase activity (35). Such variability underscores the necessity of a robust initial resection to reduce the overall tumor burden, followed by targeted PDT to address the remaining invasive cells. Ultimately, these findings provide a strong rationale for future randomized controlled trials to definitively establish the therapeutic impact of this synergistic approach compared to the current standard of care.

Our study provides also comprehensive histopathological and cellular-level characterization of the effects of 5-ALA-mediated photodynamic therapy in recurrent intracranial meningiomas. The data reveal that PDT induces a multi-mechanistic cytotoxic program, effectively targeting both the tumor matrix and the critical peritumoral niche, thereby providing a biological basis for the observed clinical outcomes.

The total ablation of progesterone receptor expression following PDT represents a significant alteration in the tumor’s biological phenotype. PR signaling is a key pathway implicated in meningioma proliferation (9). At the same time the study by Ren et al. (37) demonstrate that negative PR expression was a radiosensitive biomarker on progression free survival for *de novo* atypical meningiomas patients after gross-total resection (37). The complete loss of this receptor signature suggests that PDT inflicts profound damage that disrupts fundamental cellular identity and function, potentially pushing tumor cells towards a non-proliferative state.

The most therapeutically significant finding is the robust induction of apoptosis within the peritumoral brain tissue. The significant increase in caspase-3-positive glial cells (36.3 ± 9.6 vs. 14.8 ± 2.2 cells/mm², p<0.0001), and notably, the novel appearance of apoptosis in neuronal nuclei, provides direct evidence that PDT’s effect extends beyond the macroscopic tumor margin. Caspase-3 is a central executioner protease in the intrinsic apoptotic pathway, and its activation is a well-documented mark of PDT-induced cell death across various cancer models (38–40). The induction of apoptosis in the peritumoral zone represents a delicate balance between achieving maximal local tumor control and preserving functional brain tissue. From a biological perspective, the peritumoral microenvironment (including neuronal and glial elements) is known to undergo significant remodeling, often forming a supportive niche for residual tumor cells (41). In aggressive, recurrent meningiomas, targeting this infiltrative margin is crucial. We hypothesize that the observed apoptotic shifts in the peritumoral zone may reflect the disruption of this tumor-supportive niche, which is an expected and potentially beneficial aspect of the photodynamic effect, as long as it remains spatially restricted and clinically asymptomatic. As previously mentioned, our results showed no new or worsening neurological deficits in the experimental group postoperatively, suggesting that the observed microscopic apoptotic changes did not translate into clinical morbidity.

Our confocal microscopy data elucidates the precise subcellular chronology of PDT-induced death: initial oxidative damage leads to proteotoxic stress (Hsp70 upregulation), followed by mitochondrial dysfunction (TMRM loss), and culminates in nuclear degradation. This sequence confirms the activation of the intrinsic apoptotic pathway, initiated by massive oxidative injury. The strong induction of Hsp70 is a known cellular response to PDT-induced protein damage and serves as a clear marker of irreversible cellular stress (42). It is also worth noting the immunomodulatory antitumor effect of Hsp70, which, under stress, can be exposed on the surface of the plasma membrane of tumor cells on the one hand (43), and released into the extracellular space on the other (44). It has previously been shown that membrane-bound Hsp70 can activate the cytolytic and migratory anti-cancer activity of natural killer cells (through interaction with a CD94 receptor) (45, 46). Thus, local induction of chaperone expression can lead to stimulation of the immune response; however, this aspect was not addressed in the present work. The correlation of these profound morphological changes with the quantitative biospectroscopy data — where PpIX photobleaching served as the procedural endpoint — validates biospectroscopy as a reliable real-time surrogate for predicting ultimate biological effect.

At the same time, when analyzing morphological and immunohistochemical alterations in the areas after PDT, several potential confounding factors, such as surgical manipulation, localized ischemia, and photothermal effects, must be considered. To minimize the impact of these variables, we obtained paired biopsy specimens from the identical tumor zones strictly before and after irradiation, while the use of bipolar coagulation was avoided in these sampling areas to prevent artifactual tissue damage. Furthermore, the PDT procedure was standardized with a fixed laser power of 2.0 W and included real-time intraoperative thermometry to ensure that no significant thermal injury occurred. Although the precise independent contribution of each perioperative factor remains difficult to quantify, these methodological precautions suggest that the observed induction of apoptosis, loss of PR expression and increase Hsp70 expression are primarily associated with the photodynamic effect. Consequently, these histological shifts should be viewed as the net outcome of the integrated surgical and photodynamic intervention.

## 5 Limitations of the study

While this study provides promising evidence for the feasibility and biological activity of combined FGS and PDT in recurrent meningiomas, several limitations must be acknowledged.

First, the non-randomized design with a retrospective control group introduces potential for selection bias and confounding. Despite our efforts to ensure comparability through strict inclusion criteria and baseline analysis, unmeasured factors could have influenced the outcomes. The decision to deny randomization was primarily dictated by the exploratory and pilot nature of this research. Recurrent atypical (WHO grade 2) and anaplastic (WHO grade 3) meningiomas represent a rare and heterogeneous patient population with limited salvage treatment options. Randomized allocation was considered premature and statistically underpowered at this stage, as the primary goal was to determine if the combined intervention could be safely integrated into the neurosurgical workflow without increasing perioperative morbidity. Consequently, the observed differences in the extent of resection and recurrence rates, while encouraging, must be interpreted with caution and require validation in a randomized controlled trial. Second, the relatively modest cohort size (n=23 in the experimental group) and the single-center design of the study may constrain the external validity of our findings. The cohort, while representative of our institution’s patient population, may not fully capture the heterogeneity of recurrent meningiomas across broader clinical settings.

Third, the study is limited by a relatively short follow-up period (median 16 months; IQR 11.0–22.0) for patients in the experimental group. While preliminary clinical data were observed, the feasibility-oriented nature of this work precludes definitive conclusions regarding therapeutic superiority. Further studies with extended follow-up periods are mandatory to confirm the impact of this combined approach on long-term progression-free and overall survival.

A notable limitation of this feasibility study is the lack of a dedicated control arm utilizing 5-ALA fluorescence-guided surgery without photodynamic therapy. Consequently, it is not possible to statistically decouple the independent effect of PDT from the improved extent of resection achieved through FGS. The superior outcomes observed in the experimental group should, therefore, be viewed as a result of the integrated synergistic protocol rather than the effect of PDT as a stand-alone modality.

Finally, the quantitative biospectroscopy and PDT light delivery systems used represent a specific technological setup. The generalizability of our dosimetric parameters and fluorescence index thresholds to other hardware and software platforms requires further investigation.

Despite these limitations, this study establishes a critical foundation for future research and provides the first comprehensive histopathological evidence of PDT’s mechanism of action in meningiomas.

## 6 Conclusion

Our prospective, single-center pilot cohort study established that the integrated use of 5-ALA-guided resection and intraoperative PDT appears to be a safe and feasible strategy for managing recurrent high-grade meningiomas. Moreover, we identified and provided a solution for a key limitation of visual fluorescence assessment by validating quantitative biospectroscopy as a necessary companion for objective measurement, particularly when bridging from diagnostic fluorescence to PDT. The personalized, biospectroscopy-guided PDT protocol represents a significant evolution from fixed-dose paradigms. While our preliminary data suggest a potential improvement in local control compared to standard microsurgical resection, the exploratory nature of these comparative efficacy claims must be acknowledged. These findings justify further investigation in multi-center randomized controlled trials to definitively establish the independent therapeutic impact of PDT on long-term survival in this challenging patient population.

## Data Availability

The raw data supporting the conclusions of this article will be made available by the authors, without undue reservation.

## Glossary

5-ALA: 5-aminolevulinic acid
a.u.: arbitrary units
caspase-3: cysteinyl aspartate specific proteinase-3
DAPI: 4’,6-Diamidino-2-phenylindole
FGS: Fluorescence-guided surgery
FI: Fluorescence Index
FIS: Fluorescence Intensity Score
Hsp: Heat shock protein
MRI: Magnetic resonance imaging
PDT: Photodynamic Therapy
PpIX: Protoporphyrin IX
PS: Photosensitizer
ROS: Reactive oxygen species
TMRM: Tetramethylrodamine Methyl ester
